# Point-of-Care Tests to Strengthen Health Systems and Save Newborn Lives: The Case of Syphilis

**DOI:** 10.1371/journal.pmed.1001233

**Published:** 2012-06-12

**Authors:** David C. Mabey, Kimberly A. Sollis, Helen A. Kelly, Adele S. Benzaken, Edward Bitarakwate, John Changalucha, Xiang-Sheng Chen, Yue-Ping Yin, Patricia J. Garcia, Susan Strasser, Namwinga Chintu, Tikki Pang, Fern Terris-Prestholt, Sedona Sweeney, Rosanna W. Peeling

**Affiliations:** 1 London School of Hygiene and Tropical Medicine, London, United Kingdom; 2 Alfredo da Matta Foundation, Manaus, Brazil; 3 Elizabeth Glaser Pediatric AIDS Foundation, Kampala, Uganda; 4 National Institute of Medical Research, Mwanza, Tanzania; 5 The National Center for Sexually Transmitted Diseases, Center for Disease Control, Nanjing, China; 6 The Universidad Peruana Cayetano Heredia, Lima, Peru; 7 Elizabeth Glaser Pediatric AIDS Foundation, Lusaka, Zambia; 8 Centre for Infectious Disease Research in Zambia, Lusaka, Zambia; 9 Research Policy & Cooperation (RPC/IER), World Health Organization, Geneva, Switzerland

## Abstract

Rosanna Peeling and colleagues describe their experience of introducing point-of-care testing to screen for syphilis in pregnant women living in low- and middle-income countries.

**Summary Points**

- The widespread adoption and implementation of new diagnostic technologies in developing countries is a major challenge.
- We used a non-traditional approach to implementation research, engaging policy makers in each country in the design of a prenatal syphilis screening project using point-of-care tests and ensuring relevance to the local health care system.
- As a result of this study, all six countries changed policy to adopt point-of-care syphilis testing into their prenatal screening programmes.
- We showed that it is possible to implement a quality assurance and quality management system for point-of-care testing in all settings.
- Lessons learnt and policies implemented as a result of the project have strengthened health systems by improving access to quality-assured prenatal screening and saving newborn lives.

## The Challenge

Poor rural populations in low- and middle-income countries (LMICs) do not have access to diagnostic laboratories. Until recently, this has meant that they could not be screened for asymptomatic infections such as HIV or syphilis, and could only be treated syndromically when presenting with symptoms such as fever. This situation has now changed. Simple, rapid, and affordable point-of-care tests (POCTs), which do not require electricity, a laboratory, or highly trained staff, are now available and widely used for several common infections in LMICs [1]. POCTs offer an unprecedented opportunity to reduce inequalities in health, and to help LMICs achieve the health-related Millennium Development Goals (MDGs) [2].

Syphilis in pregnancy causes more than half a million stillbirths or neonatal deaths annually [3],[4]. These deaths could be prevented if all pregnant women were screened for syphilis and treated with a single dose of penicillin before the third trimester [5]. Screening of pregnant women for syphilis is recommended policy in almost every country, and even in LMICs the vast majority of women attend antenatal clinic (ANC) at least once during pregnancy; yet in sub-Saharan Africa, it has been estimated that fewer than 40% of those who attend ANC are screened for syphilis [4]. Why is this? The rapid plasma reagin (RPR) test, which is used for screening in many countries, is only available in laboratories, as reagents require refrigeration and the reactions are often batched. Blood is often sent to a central laboratory, or the tests are performed at the end of the day, requiring patients to return for their results. In one study in Nairobi, fewer than 10% of women who tested positive received treatment [6].

New POCTs for syphilis are now available and some of them meet the ASSURED criteria, being Affordable, Sensitive, Specific, User-friendly, Rapid and robust, Equipment-free, and Deliverable to those who need them [7]. However, for POCTs to maximize their potential, a number of challenges must be addressed. Health care workers (HCWs) must be properly trained in how to use them and how to interpret their results; the quality of the tests and the testing must be maintained through a quality assurance programme; and supply chains must be improved to ensure that POCTs and effective treatment are continuously available. Here we describe our experiences in attempting to overcome these challenges and introduce syphilis POCTs to prevent adverse outcomes of pregnancy in six LMICs. Our goal was to determine the feasibility of introducing POCTs into different settings in countries with different health systems and cultural and socioeconomic contexts. We measured several health systems indicators to determine whether we met the success criteria as shown in Table 1.

**Table 1:** Health systems indicators and proposed success criteria.

Health System Indicators	Outcome Measures	Success Criteria
Coverage (increase in access to screening)	# women pregnant screened pre- and post-POCT introduction	90% coverage post-POCT introduction
Improved health outcome	# infected women treated	90% of women who tested positive treated
Acceptability by clients and health workers	Job satisfaction; client satisfaction with service	Increased job satisfaction and client satisfaction with services
Quality assurance	# workers who passed proficiency test	90% of CHWs passed the proficiency test
Integration into existing programmes	Effect on ANC and prevention of mother-to-child transmission (PMTCT) programmes for HIV	Synergy with existing programmes
Sustainability	# countries which change policy	Policy change, development of national guidelines for use, and plans for scale up

## The Project

Project sites were located in countries where national guidelines recommend antenatal screening for syphilis. We convened a workshop with all the principal investigators (PIs) at the start of this project to develop a set of guiding principles and a generic protocol for all countries to use as a template.

### Policy and Stakeholder Consultations

Each country project team consulted with national and local health authorities and other stakeholders on the design and location of the project, and sought their endorsement of the strategy of same-day testing and treatment (STAT) for syphilis. All site protocols were approved by the relevant institutional review boards (IRBs) and the World Health Organization (WHO) Ethics Review Committee.

### Project Settings and Study Populations

Syphilis POCTs were introduced in rural antenatal clinics in Tanzania, Uganda, and China; and in both rural and urban clinics in Peru and Zambia. In Brazil, community-based screening was introduced in remote indigenous populations where there were no fixed health facilities or laboratories, and no screening was previously available. The opportunity was taken to screen all sexually active individuals (Table 2).

**Table 2:** Key indicators for syphilis screening before and after POCT introduction.

Country and Population Screened	Screening Method Pre-POCT	% People Screened Pre-POCT	% People Screened Post-POCT	% Positive Post-POCT	% People Treated Post-POCT
Amazonas region, Brazil (sexually active population)	No screening available	No screening available	45,971/84,038 (54.5% )	Sexually active: 745/45,971 (1.62%); pregnant women^a^: 64/4,695 (1.36%)	808/808 (100%)
Rural ANC in Guangdong province, China	TRUST (RPR) confirmed with TPPA	N/a^b^	5,272/5,489 (96.0%)	109/5,272 (1.9%)	102/109 (93.6%)
Maternity hospital in Lima and ANCs in Callao, Peru	RPR	9,595/18,757 (51%)	15,985/16,839 (95%)	146/15,985 (1.0%)	128/134^c^ (97%)
District Hospital and 51 health facilities in Geita District, Tanzania	RPR	634/3,561 (17.8%)	58,249/58,249 (100%)	6,345/58,249 (10.9%)	5,717/6,345 (90.1%)
Kampala Hospital and rural ANCs in Uganda	RPR	140/8,475 (1.7%)	13,131/14,540 (90.3%)	690/13,131 (5.3%)	715/690 (103.6%)^d^
Lusaka Hospital and rural ANCs in Mongu district, Zambia	RPR	12,761/15,967 (79.9%)	11,460/11,985 (95.6%)	1,050/11,460 (9.2%)	1,000/1,050 (95.2%)

a Pregnant women were a subset of the sexually active population.

b N/a: No data available before the introduction of POCTs as testing was done by a laboratory and these services were not widely available in rural China.

c Twelve POCT-positive pregnant women had a previous history of syphilis and were either RPR negative or had received treatment recently. The treating physicians did not think additional treatment was required.

d ANC attenders whose accompanying partners were found to have syphilis were offered presumptive treatment even if their own result was negative. This resulted in more women being treated than had positive tests.

RPR, rapid plasma reagin test; TPPA, *Treponema pallidum* particle agglutination assay.

Country-specific testing and treatment algorithms and the test used for the project were selected by each country in consultation with the Ministry of Health (MOH). Brazil, Peru, Tanzania, Uganda, and Zambia used the SD Bioline Rapid Syphilis test (Standard Diagnostics, Seoul, Korea), which we had previously evaluated and found to have acceptable performance [7] and was available through the WHO Bulk Procurement Programme at negotiated pricing. In China the Rapid Syphilis test (Wantai Ltd, Beijing, China) was used.

### Baseline Survey

In each country, published data and MOH reports on the prevalence of syphilis and the coverage of screening and treatment in ANCs were reviewed. Except in Brazil, where no testing had previously been available for remote indigenous populations, a baseline survey was undertaken, at which project sites were visited, HCWs were interviewed concerning current practice for syphilis screening and barriers to achieving full coverage, and recent records on the number and proportion of women screened for syphilis were reviewed. Where coverage was sub-optimal, reasons for this were elicited from HCWs and their supervisors.

### Site Preparation: Training, Quality Assurance, and Supply Chain Management

HCWs were trained in the use of syphilis POCTs, using training materials developed for the project, translated into the local language, and adapted according to the local cultural and social context. Courses for trainers and supervisors included practical training on how to perform the tests and interpret the results, and how to train HCWs and assess their competence, with additional training in stock management, record keeping, and quality control.

For quality assurance, supervisors were provided with proficiency panels prepared by a reference laboratory, consisting of tubes in which sera characterized as positive or negative had been allowed to dry at room temperature. Dried sera were reconstituted with buffered saline on site and used to assess the performance of POCTs by HCWs. This simple, low-cost method was originally developed to assure the quality of HIV testing [8]. Syphilis POCTs were provided to health facilities through the normal supply chain to allow the PIs to monitor supply chain problems and provide sustainable solutions in case of stock-outs. All individuals who tested positive at each site were treated with the first dose of benzathine penicillin on the same day.

### Continuous Quality Improvement, Outcome Measures, and Success Criteria

An internal monitor was appointed in each project team to monitor quality and outcome measures continuously so that problems were brought to the attention of the PIs and regional medical officers, and solutions were tried until a sustainable solution was found. External monitors visited the sites and provided suggestions for further improvement. The opinions of HCWs on the ease of use and acceptability of the POCTs were sought through interviews and focus group discussions. The results of the baseline survey and interim data were shared with national and regional policy makers and programme managers through dissemination workshops at each site. Data on coverage of screening and treatment and on HCWs' experiences were collected using standard data collection instruments, double entered, and analysed in country. The results from each country are being analysed in more detail and will be submitted for publication separately.

## Results

### Impact of POCTs on Coverage of Syphilis Screening and Treatment

Over 100,000 pregnant women were screened for syphilis. The introduction of POCTs increased the proportion of ANC attenders screened for syphilis to >90%, and the proportion of pregnant women with syphilis who were treated the same day exceeded 90% in all countries (Table 2). In Brazil, HCWs in remote communities succeeded in screening 55% of the sexually active population (defined as ≥10 years of age) for syphilis, exceeding the 30%–40% target originally set.

### Acceptability of POC Syphilis Tests among HCWs and Study Populations

POCTs were well accepted, and considered easy to perform, by HCWs at all sites. For example, in Uganda, 82% of HCWs described the POCTs as “very easy to perform.” At sites where RPR had been previously performed, HCWs reported that they could test more patients per day with the POCT. Clients at all sites appreciated receiving results and treatment at their first visit, rather than being asked to return for their results as they had in the past. Most clients at all sites preferred a finger prick to venepuncture because of the smaller volume of blood required.

Laboratory staff in the main maternity hospital in Peru were initially opposed to the idea of nurses and midwives doing a task that had traditionally been done by laboratory technicians, and the hospital management did not believe it was necessary to introduce POCTs. Although coverage of screening and treatment for syphilis at the ANC was high before the introduction of POCTs, women did not receive test results for at least 15 days [9]. Moreover, a significant number of women who delivered in the hospital had not attended ANC and, among women admitted with early or mid-term abortions, few were screened. When shown these data, the hospital director welcomed the introduction of POCTs. Laboratory staff trained and supervised the nurses and midwives doing the tests, and set up a quality assurance programme using a proficiency panel. This programme was enthusiastically approved by both laboratory staff and nurses, each of whom received a certificate if they performed well on the proficiency panel.

The indigenous population in Brazil initially had reservations about the POCT because of the possibility of a positive test result, the pain caused by the finger prick, and concerns about the treatment. In rural areas of China, pregnant women of ethnic minorities were less likely to accept the test due to the low perception of risk. This gradually changed as the programme became more established and expanded into the community.

### Experience with Quality Assurance

**Figure 1:**
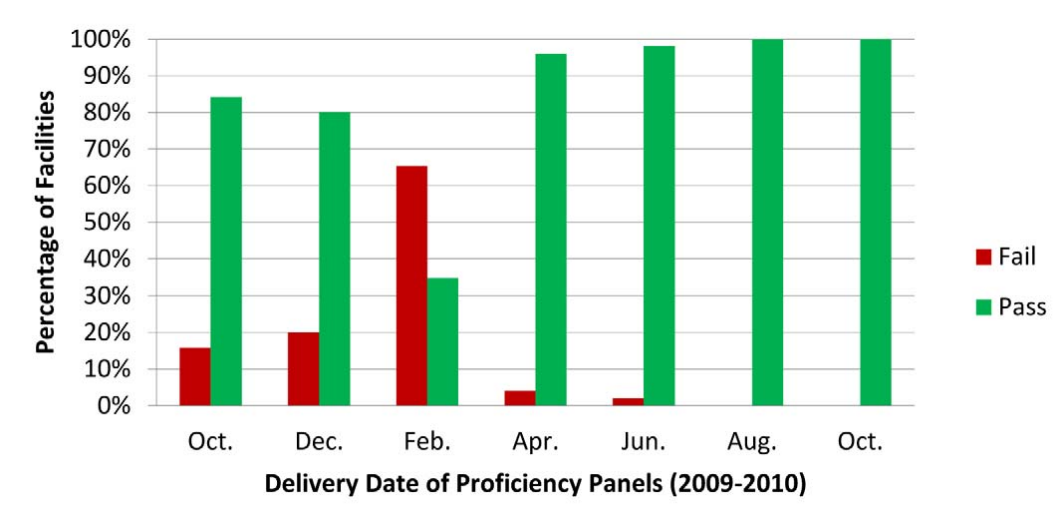
Percentage of facilities receiving a passing* or failing score on each of the seven proficiency panels. *Pass defined as ≥67% score on the proficiency panel.

The proficiency panel using dried tube specimens allowed the study team to identify HCWs in need of refresher training. Figure 1 shows the results obtained in health facilities in Geita District in Tanzania over an 18-month period, using a panel consisting of six tubes containing a mixture of positive and negative samples. Six months after the initial training workshop, the percentage of facilities failing the proficiency panel rose to 65%. In several of these clinics, the staff member performing the tests was not the ones who had been trained to do so. In response to the poor performance across the district, the laboratory coordinator arranged for one staff member from each of the failing health facilities to attend a refresher training workshop. HCWs were instructed to re-train colleagues upon return to their facility. Following the refresher training, proficiency panel scores improved greatly.

### Integration into PMTCT Services for HIV

In Uganda and Zambia, we assessed the impact of introducing syphilis POCTs on existing prevention of mother-to-child transmission (PMTCT) programmes for HIV (Table 3). After POCT introduction, there was a significant increase in ANC attendance, resulting in more pregnant women being tested for HIV in both countries. In Zambia, there was also a significant increase in the number of women receiving anti-retroviral (ARV) prophylaxis and referred to care and treatment following the introduction of syphilis POCTs. We believe that increased awareness of the importance of syphilis and HIV in pregnancy, better supportive supervision, and greater efficiency of integrated services all contributed to this improvement.

**Table 3:** Effect of introducing POCTs for syphilis on PMTCT of HIV programmes in Uganda and Zambia.

Outcome Measure	Uganda	Zambia
HIV testing pre-POCT (%)	95.6% (*n* = 6,479)	95.5% (*n* = 7,479)
HIV testing post-POCT (%)	96.4% (*n* = 11,192)	97.7% (*n* = 11,414)
*p*-value	*p* = 0.009	*p*<0.0001
ARV prophylaxis pre-POCT (%)	78.5% (*n* = 570)	98.3% (*n* = 1,303)
ARV prophylaxis post-POCT (%)	83.6% (*n* = 964)	100.0% (*n* = 2,036)
*p*-value	*p* = 0.007	*p*<0.0001
Refer to care & treatment pre-POCT (%)	16.9% (*n* = 85)	73.7% (*n* = 977)
Refer to care & treatment post-POCT (%)	16.1% (*n* = 118)	84.6% (*n* = 1,721)

### Impact on Health Policy

This project has brought to the forefront the power and value of implementation research in bringing about rapid policy change and strengthening health systems. It also has broader implications for health systems strengthening, which is fundamental to the achievement of the health-related MDGs. The inclusion of policy makers from the beginning of the project, and keeping them informed during POCT implementation, led to policy change even before the research was completed. In all six countries, regular meetings were held between project staff and health authority officials at national, regional, and district levels, focussing on those responsible for both maternal and child health (MCH) and HIV/AIDS programmes. This is important, as previous studies have found that it is often not clear whether antenatal syphilis screening is the responsibility of the MCH, HIV/AIDS, or sexually transmitted infection (STI) control programmes [4],[10].

All six countries changed their policy to recommend the use of rapid tests to provide a STAT service. National guidelines have been amended accordingly. Meetings jointly sponsored by our programme and the WHO have been held with policy makers from many countries in Asia, Africa, and South America, at which we shared the toolkit developed for test introduction and presented project results. This has resulted in many more countries in all three regions adopting and rolling out the use of POCTs for syphilis screening.

In Brazil, as a result of this study, the minister of health announced in 2011 that syphilis screening for remote populations would be one of the three main priorities for the national programme for the control of HIV and STIs, and the president announced the “Stork” Initiative to bring healthy babies free of both HIV and syphilis. In China, Premier Wen Jiabao chaired a meeting in November 2010 at which he announced that a screening policy for syphilis would be introduced in ANCs in China [11]. The WHO has now included the use of POCTs for syphilis in its STI Management Guidelines. Our project leaders have worked with their national MOH to develop national guidelines for the use of POCTs in prenatal screening, and are now involved in scaling up training and quality assurance for neighbouring countries as well.

## Discussion

This project has shown that POCTs for syphilis can be effectively introduced in a range of settings, from cities in China and Peru, to remote villages in East Africa, and even more remote indigenous populations in the Amazon rain forest. By working with the existing health care system to integrate testing, the introduction of POCTs resulted in large numbers of women being tested and treated for syphilis, averting many stillbirths and reducing neonatal mortality. Importantly, the outcomes resulted in policy change in all project countries to incorporate POCTs into national guidelines. The project has highlighted the drivers for success as well as the key challenges that need to be overcome to scale up the use of POCTs. Their introduction could have a major impact in reducing inequalities in health care and motivating HCWs, who are happy to be able to provide a diagnostic service to their patients that results in immediate treatment. Unlike the financial motivation provided by many vertical programmes, this type of motivation through increased job satisfaction has no adverse impact on other services and is more sustainable.

The introduction of POCTs can serve as a “probe” for the efficient functioning of a health system more broadly, as the intention to introduce POCTs (a “technology”) for syphilis had “knock-on” effects on other important “building blocks” of the health system including service delivery, the health workforce, health information, health financing, and leadership and governance [12]. It represents a real and powerful example of the need for “systems thinking” [13], an approach to problem solving that views “problems” as part of a wider, dynamic system and which demands a deeper understanding of the linkages, relationships, interactions, and behaviours among the elements that characterize the entire system.

This project has highlighted the importance of quality assurance programmes for POC testing. Without them, failures in HCW proficiency would have gone unnoticed. It is deeply concerning that most HIV testing programmes in developing countries now rely on POCTs, but have no quality assurance programme to ensure that the tests are properly performed [14]. The continuing use of proficiency panels to monitor the quality of testing is particularly important where there is a rapid turnover of staff.

In spite of the success of this project in increasing access to syphilis screening in many countries, a number of challenges remain. POCTs cost a little more than the RPR test, but are available through the WHO bulk procurement programme for less than US$1. The cost per woman screened ranged from US$1.9 to $6.1 in Tanzanian health facilities [15], and screening pregnant women for syphilis remains one of the most cost-effective health interventions [16]. HCWs and their supervisors received training in stock management, but problems remain at the national level, and stock outs of tests and treatments still occurred. One visit to Geita District at the end of the project found that ten of 17 facilities had recorded at least one day of stock out of POCTs over a 4-month period.

### Limitations of This Study

We were not able to show impact, as we were funded only to determine feasibility of POCT introduction for one year. We adopted a non-traditional approach to implementation research since we wanted to determine feasibility in a variety of cultural and socioeconomic settings. We decided not to have a rigid protocol to allow countries to improvise as necessary to maximize POCT implementation based on STAT. However, we did ensure a uniform set of outcome measures were collected across all sites, including quality assurance of POC testing. We used policy change as an indicator of success, but strengthening fragile health systems requires sustained effort of which policy is only a start.

## Moving Forward

The project has implications for improved health care delivery beyond the diagnosis and management of syphilis in pregnancy. Every case of congenital syphilis represents a failure of health care delivery; attempts to prevent it could act as a “window of knowledge” or “proxy indicator” for the robustness of health service delivery systems more generally. Lessons learnt about appropriate technologies, supply chain management, training and motivating HCWs, quality assurance, effective teamwork, and open communication are valuable not just for syphilis, but for health care delivery, and can ultimately lead to sustained health systems strengthening. There is even a potential for “reverse innovation” where improved, cost-effective processes discovered in the developing world are adopted by health care delivery organizations in industrialized countries.

We wrote about the tragedy of babies avoiding HIV and dying of syphilis because of vertical disease control programmes and compartmentalized health care infrastructures [17]. Our long-term vision is to facilitate the development and implementation of an essential POCT package for prenatal care that could be broadened to include diagnostics for both infectious diseases and conditions such as anaemia and pre-eclampsia to ensure safe motherhood and healthy babies. Such an integrated approach could result in improved efficiency, a more robust health care system, and lives saved.

## Abbreviations

ANC: antenatal clinic
ARV: anti-retroviral
HCW: health care workers
LMIC: low- and middle-income country
MCH: maternal and child health
MDG: Millennium Development Goal
MOH: Ministry of Health
PI: principal investigator
PMTCT: prevention of mother-to-child transmission
POCT: point-of-care test
RPR: rapid plasma reagin
STAT: same-day testing and treatment
STI: sexually transmitted infection
WHO: World Health Organization
